# Heart rate variability as predictor of mortality in sepsis: A prospective cohort study

**DOI:** 10.1371/journal.pone.0180060

**Published:** 2017-06-27

**Authors:** Fábio M. de Castilho, Antonio Luiz P. Ribeiro, José Luiz P. da Silva, Vandack Nobre, Marcos R. de Sousa

**Affiliations:** 1 Hospital das Clínicas and School of Medicine, Universidade Federal de Minas Gerais (UFMG), Belo Horizonte, Brazil; 2 Department of Statistics, Universidade Federal de Minas Gerais (UFMG), Belo Horizonte, Brazil; 3 Núcleo Interdisciplinar de Investigação em Medicina Intensiva (NIIMI), UFMG, Belo Horizonte, Brazil; University of Florida, UNITED STATES

## Abstract

**Background:**

Sepsis is a serious medical condition with increasing prevalence and high mortality. The role of the autonomic nervous system in pathophysiology of sepsis has been increasingly researched. The objective of this study is to evaluate the Heart rate variability (HRV) as a predictor of mortality in septic patients.

**Methods:**

This was a prospective cohort of patients diagnosed with sepsis. Patient recruitment was carried out at ICU in tertiary university hospital between March 2012 and February 2014. Clinical data and laboratory exams were collected at admission. Each patient underwent a 20-minute Holter and a 24-hour Holter on the first day of enrollment. The primary outcome was the 28-day all-cause mortality.

**Results:**

A total of 63 patients were included. Patients were categorized into nonsurvivor group (n = 16) or survivor group (n = 47) depending on this endpoint. Survivors were younger (48.6 years vs. 63.0 years), had better renal function and lower values in severity scores (APACHE II and SOFA) compared to nonsurvivors. In the 20-minute Holter, SDNN, Total Power, VLF Power, LF Power and LF/HF of nonsurvivors were significantly lower than those of survivors (p = <0.001, p = 0.003, p = 0.002, p = 0.006, p = 0.009 respectively). ROC curve of SDNN was built, showing area under the curve of 0.772 (0.638–0.906) for mortality. The value of 17ms was chosen as best SDNN cutoff to discriminate survivors and nonsurvivors. In the Cox proportional regression, adjusted for SOFA score and for APACHE II, a SDNN ≤ 17ms was associated with a greater risk of death, with hazard ratios of 6.3 (1.4–28.0; p = 0.015) and 5.5 (1,2–24,8; p = 0.027), respectively. The addition of the dichotomized SDNN to the SOFA model reduced AIC and increased the concordance statistic and the R^2^, indicating that predictive power of the SDNN + SOFA model is better than predictive power of SOFA only.

**Conclusions:**

Several HRV parameters are reduced in nonsurviving septic patients. SDNN ≤17 is a risk factor for death in septic patients, even after adjusting for severity scores.

## Introduction

Sepsis is a serious medical condition which prevalence has increased significantly in recent decades[1], making 31.5 million new cases to be expected in hospitals around the world each year[2]. Due to the high mortality associated with this condition, which can reach 48.6%[3], it is essential to search risk factors for death and predictive scoring systems to help clinical decision in septic patients. Predictive scoring systems such as APACHE II (Acute Physiology and Chronic Health disease Classification System II), SOFA (Sepsis-related Organ Failure Assessment), SAPS-3 (Simplified Acute Physiology Score III) and MODS (Multiple Organ Dysfunction *Score)* combine clinical and laboratory characteristics to assess the severity of illness. However, none of these scores considers in its composition changes in the autonomic nervous modulation caused by sepsis.

Heart rate variability (HRV) is a noninvasive indirect test to evaluate autonomic function[4, 5]. In normal situations, heart rate varies, indicating the heart's capacity to adapt to different situations. HRV measures the oscillation of the intervals between consecutive heart beats, which are related to, the influences of the autonomic nervous system on the sinus node[6]. Patients with sepsis have reduced HRV compared to healthy patients, as demonstrated in small studies[7–9]. Furthermore, HRV parameters such as low frequency (LF) power are positively correlated with APACHE II and SOFA[10] and negatively correlated with interleukins[11]. Small studies have suggested that sepsis survivors present HRV parameters (e.g., standard deviation of NN interval, SDNN) higher than nonsurvivors[12, 13]. However, no study has defined a specific HRV parameter and a cut-off point that can be used in practice for the prediction of the risk of death in septic patients. Thus, the use of HRV as an independent predictor of death in sepsis deserves further investigation.

The objective of this study was to evaluate the role of HRV—recorded both with the 20 minute and the 24 hour-Holter—as a predictor of death in patients with severe sepsis, defined by the presence of infection, the Systemic Inflammatory Response Syndrome criteria and evidence of organ dysfunction.

## Materials and methods

### Study design

This was a prospective cohort of patients diagnosed with severe sepsis. This report follows "Strengthening the Reporting of Observational studies in Epidemiology", the STROBE Statement[14].

### Patient population

Patient recruitment was carried out at one of the Intensive Care Units of Hospital das Clínicas of the Universidade Federal de Minas Gerais (ICU-UFMG), Brazil, a mixed ICU with eight beds. From March 10^th^, 2012 to February 06^th^, 2014, all adult (i.e., 18 year-old or older) patients, hospitalized in the ICU-UFMG that had suspicion of sepsis at admission or during the ICU stay, and at least one organ dysfunction supposedly related to the infectious condition were considered for potential eligibility. Sepsis was defined according to the Sepsis 2 Consensus[15] as being a Systemic Inflammatory Response Syndrome associated with a confirmed infection or strongly suspected infection. Systemic Inflammatory Response Syndrome was defined as the presence of at least two of the following: 1- Body temperature higher than 38°C or lower than 36°C; 2- Heart rate higher than 90/min, 3- Hyperventilation evidenced by respiratory rate higher than 20/min or PaCO2 lower than 32 mmHg; 4- White blood cell count higher than 12,000 cells/μl or lower than 4,000/μl or at least 10% of immature forms [16]. The presence of at least one organ dysfunction was based on severe sepsis definition of Surviving Sepsis Campaign[17]. Despite inclusion phase of this study was conducted prior to publication of the Sepsis 3 definitions[18], all included patients met the *criteria* for *Sepsis* proposed in this consensus.

Exclusion criteria were: moribund patients (death previewed for the next 24 hours), patients with proposal for exclusive palliative care, septic patients under antibiotic therapy for more than 48 hours prior to enrollment and patients with non-sinus rhythm or with pacemaker.

### Study protocol and general procedures

This study was approved by the Ethics Research Committee of the Universidade Federal de Minas Gerais, Brazil, and all included patients or their family members signed a written informed consent. Clinical data was collected at admission and during the clinical follow-up of patients through a dedicated Clinical Report Form. The main variables collected were: age, gender, comorbidities, main diagnosis at the time of inclusion, primary site of infection and microbiological findings, antibiotic used, Sepsis related Organ Failure Assessment score (SOFA)[19] and Acute Physiology And Chronic Health Evaluation II (APACHE II)[20], both evaluated at the time of inclusion.

### Heart rate variability analysis

Each patient enrolled in the study underwent a 3-channel Holter (Cardios^®^ CardioLight model, São Paulo, Brazil) on the first day of enrollment. Two recordings were made sequentially: 20 minutes record and 24 hours record. Both Holter monitors were placed and removed from the patients by one of the medical researchers. The first measure (20 minutes record) was made with the patient in supine position and no intervention (nursing, physiotherapy, etc.) was made during its recording. The 24-hour measure was made without interference in the normal ICU care routine. Data analysis to derive HRV was performed using system specifically developed for this purpose (Cardios^®^), which automatically calculates the following indices of HRV in the time domain: Normal-to-Normal (NN) average interval, standard deviation of the NN interval (SDNN), square root of the squared mean of the difference of successive NN-intervals (r-MSSD), percentage of NN intervals deviated by more than 50 ms from adjacent NN-intervals (pNN50); and frequency domain with fast Fourier Transform (FFT) method: Total Power, Very low frequency power (VLF Power), Low frequency Power (LF Power), High frequency power (HF Power) and Ratio between LF and HF (LF/HF). In the 24-hour Holter, HRV analysis was performed only in the time domain. We have performed manual review of all Holter’s automatic interpretation, including the rhythm and the complexes recorded (e.g., normal QRS, ventricular extrasystoles, supraventricular extrasystoles, tachycardia, bradycardia, artifacts etc.). Artifacts and irregular beats (extrasystoles, noise and missing beats) were manually deleted before HRV analyses. In the 24-hour Holter, HRV in the time domain was calculated over an entire 24-hour period. In the 20 minutes Holter, HRV was calculated both in the time domain and in the frequency domain over the entire first 10 minutes of recording.

### Outcomes

The primary outcome of this study was the all-cause mortality at 28 days of follow-up. Patients were categorized into nonsurvivor group or survivor group depending on the primary endpoint. Several HRV parameters were compared between these two groups.

### Sample size

The sample size calculation tested the hypothesis that SDNN distribution would be the same between surviving and nonsurviving patients. The statistical test used was the nonparametric Mann-Whitney that assumes that the data is measured at least in ordinal scale. The formulas adopted for sample size calculation are described in Zhao, Rahardja, & Qu[21] and implemented in software R[22]. A pilot sample constituted by the first twenty patients included in the study (6 deaths and 14 survived) was considered to estimate the parameters required to calculate the final sample size. Tertiles of SDNN were calculated from this pilot sample, defining three ranges. The proportion of subjects in each of these three ranges was obtained. Keeping the allocation ratio (i.e., survivors and non survivors) similar to that observed in the pilot study, a requirement of 58 patients (44 survivors and 14 deaths) was defined for the final analysis. We considered a type I error of 5% and 80% power.

### Statistical analysis

The normality of each continuous variable was assessed by means of the Kolmogorov-Smirnov test. Data obtained from continuous variables are expressed as either mean and standard deviation if they have normal distribution, or median and interquartile range (25th and 75th percentiles) if they have non-normal distribution. Data concerning categorical variables are expressed as absolute numbers and proportions. Clinical characteristics of survivors and nonsurvivors were compared using Student *t* test, Mann–Whitney test and exact Fisher test according to the type and the distribution of the variable. In order to choose the best cut-off point of SDNN, a ROC curve was used having death as the reference and SDNN as the parameter test[23]. Then the dichotomized SDNN was used to build Kaplan–Meier survival curves, and they were compared by log-rank test. The influence of HRV variables on survival was studied with Cox regression. Considering the small sample, we used modeling strategies to avoid the risk of overfitting and the excessive “optimism” of the model [24]. The calibration of the models with only SOFA and with dichotomized SDNN + SOFA was assessed with the the Grønnesby and Borgan test (GF Test). The performance of those prediction models was assessed using concordance measure, Explained variation (R^2^) and Akaike Information Criterion (AIC). In order to evaluate the reclassification of the SOFA + SDNN model compared to the SOFA model only, we set the time of 28 days for the predictions of the risks, and calculated continuous net reclassification improvement (NRI) and integrated discrimination improvement (IDI). A p < 0.05 was considered statistically significant for all analyses.

All the statistical analyses were conducted in SPSS version 23 (SPSS Inc., Chicago, IL, USA) and R version 3.3.0 (The R Foundation for Statistical Computing)[22] using the packages *rms*, *survMisc and survIDINRI*.

## Results

**Fig 1:**
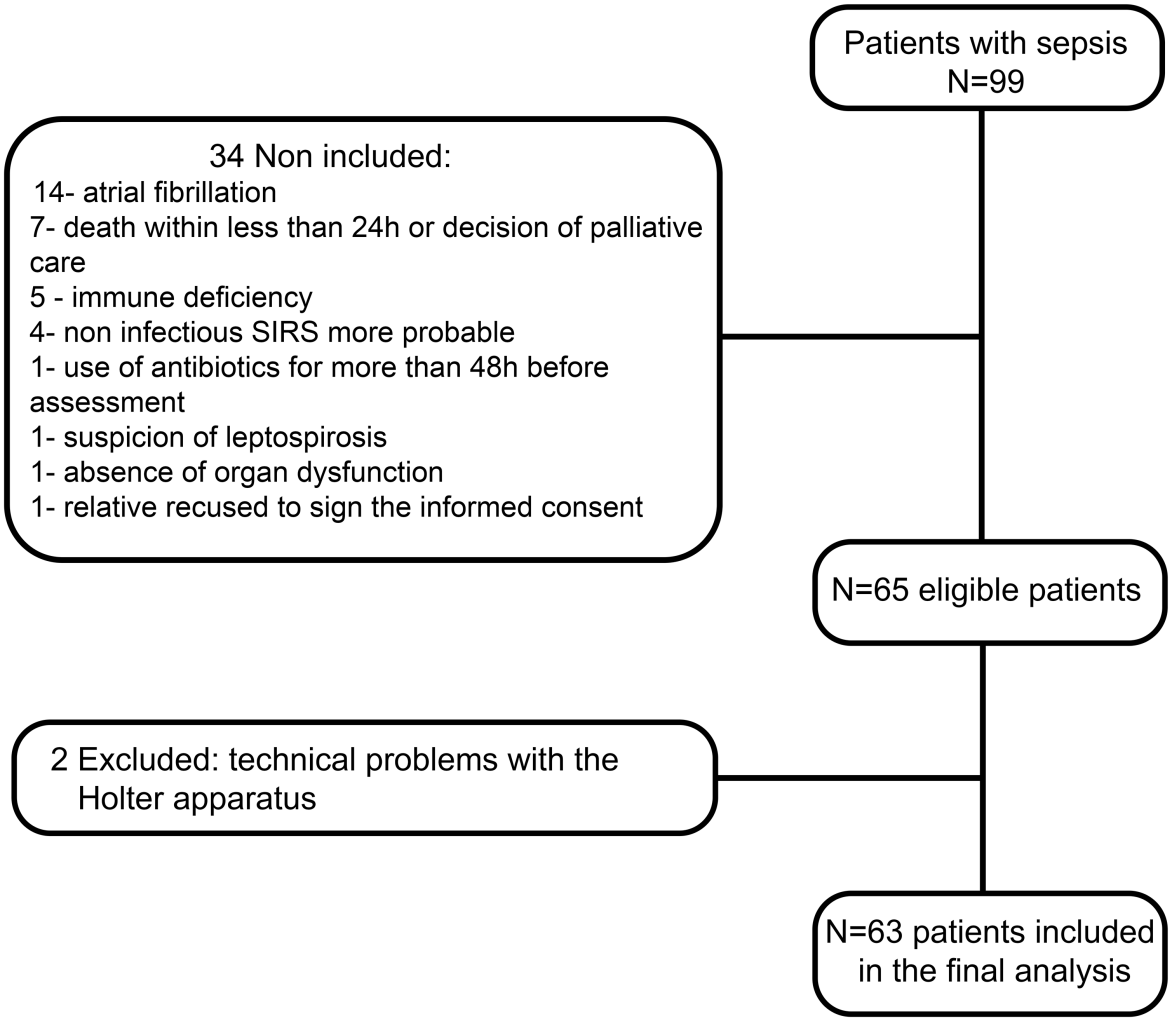
Flowchart of study procedures.

From a total of 99 patients with sepsis assessed during the study period, 79 patients were initially identified as eligible. Of these, two patients were excluded because of technical problems with the Holter equipment and 14 patients were excluded due to atrial fibrillation. Thus, 63 patients were included in the final analyses (Fig 1).

The baseline characteristics of the included patients are shown in Table 1, stratified according to the 28-day all-cause mortality. As presented, 16 (25.4) out of the 63 patients died during the follow-up of 28 days. Survivors were younger (48.6 years vs. 63.0 years), had better renal function and lower values in severity scores (APACHE II and SOFA) compared to nonsurvivors. There were no significant differences in other baseline characteristics.

**Table 1:** Baseline characteristics of the patients.

Attribute	Survivors (n = 47)	Nonsurvivors (n = 16)	p-Value
Age (y), SD	49 (17.8)	63 (17.9)	0.007
Male gender, %	27 (57.4)	11 (68.8)	0.425
APACHE II, SD	14.15 (5.93)	21.94 (8.45)	<0.001
SOFA, SD	6.91 (2.84)	10.56 (4.21)	0.004
Mechanical Ventilation, %	24 (51.1)	12 (75.0)	0.095
**Underlying disease, n (%)**			
Cirrhosis	2 (4.3)	1 (6.2)	0.896
Dialytic patients	4 (8.5)	1 (6.2)	0.773
Hypertension	18 (38.3)	9 (56.2)	0.369
Diabetes	11 (23.4)	4 (25.0)	0.354
Stroke	7 (14.9)	2 (12.5)	0.793
Peripheral arterial disease	1 (2.1)	0 (0.0)	0.801
Heart Failure^b^	4 (8.5)	3 (18.8)	0.459
Coronary artery disease	4 (8.5)	3 (18.8)	0.288
Neoplasia	4 (8.5)	2 (12.5)	0.541
Chronic Obstructive Pulmonary Disease	3 (6.4)	0 (0.0)	0.453
Smoking	13 (27.7)	2 (12.5)	0.112
**Laboratory data**			
Hemoglobin (g/dL)	10.0 (1.86)	10.4 (3.15)	0.582
White blood cells (per mm3)	16171 (9653)	17929 (10005)	0.535
Platelet × 10^3^	228 (121)	187 (116)	0.240
Lactate^a^ (mmol/L)	1.80 (1.6–4.0)	2.55 (1.6–4.2)	0.059
C-reactive protein (mg/L)	229 (115)	287 (109)	0.082
Urea (mg/dL)	55.4 (34.4)	107.3 (47.4)	<0.001
Creatinine^a^ (mg/dL)	0.72 (0.49–1.6)	2.28 (0.96–2.85)	0.004
Creatinine clearance^a^ (mL/min)	108 (58–157)	29 (18–86)	0.005
Glucose (mg/dL)	144 (56)	171 (99)	0.176
International normalized ratio	1.2 (1.1–1.4)	1.4 (1.2–2.0)	0.026
**Infection source, n (%)**			
Respiratory tract	16 (34.0)	7 (43.8)	0.687
Intra-abdominal	8 (17.0)	4 (25.0)	0.737
Urinary tract	5 (10.6)	1 (6.3)	0.990
Catheter	7 (14.9)	2 (12.5)	0.860
Soft tissue	3 (6.4)	1 (6.3)	0.563
Central Nervous System	0 (0.0)	1 (6.3)	0.561
Undetermined	7 (14.9)	0 (0.0)	0.239
Miscellaneous	1 (2.1)	0 (0.0)	0.561

Data presented as mean (SD), median (interquartile range) or absolute number (percentage).

^a^ = variables with non-normal distribution;

^b^ = Heart Failure was defined as previous echocardiogram with ejection fraction ≤ 50%.

HRV measures of each group are listed in Table 2. In 20-minute Holter, SDNN, Total Power, VHF Power, LF Power and LF/HF of non-survivors were significantly lower than those of survivors. There was no statistically significant difference in HRV measured in the 24 hours Holter between the two subgroups.

**Table 2:** Heart rate variability measures.

Parameter	Survivors (n = 47)	Nonsurvivors (n = 16)	p-Value
**20 Minutes Holter**			
Artifacts and irregular beats^a^ (%)	2.0 (1.0–5.3)	2.5 (0.3–8.0)	0.112
Day recordings^a^^,^^b^, %	37 (78.7)	13 (81.3)	0.829
NN (ms)	658.2 (166.9)	606.0 (130.4)	0.261
SDNN (ms)^a^	19.0 (10.0–36.0)	8.5 (5.0–14.5)	<0.001
rMSSD (ms)^a^	9.0 (6.0–28.0)	7.5 (6.0–12.8)	0.199
pNN50 (%)^a^	0.13 (0.00–4.73)	0.14 (0.00–0.63)	0.482
Total Power (ms^2^)^a^	136.0 (46.0–590.0)	24.0 (5.0–173.5)	0.003
VLF Power (ms^2^)^a^	90.0 (27.0–243.0)	9.5 (2.5–72.5)	0.002
LF Power (ms^2^)^a^	18.0 (6.0–83.0)	2.0 (1.0–24.0)	0.006
HF Power (ms^2^)^a^	9.0 (5.0–51.0)	6.5 (2.3–57.0)	0.343
LF/HF^a^	1.29 (0.47–3.63)	0.40 (0.21–1.84)	0.009
**24-Hour Holter**			
Artifacts and irregular beats^a^ (%)	1.0 (1.0–1.0)	1.0 (1.0–2.3)	0.955
NN (ms)	661.0 (133.4)	622.9 (123.5)	0.345
SDNN (ms)	58.2 (39.4)	50.7 (24.5)	0.402
rMSSD (ms)^a^	14.0 (8.0–28.3)	15.5 (10.0–29.3)	0,944
pNN50 (%)^a^	0.55 (0.05–3.11)	0.66 (0.24–2.78)	0,688

NN = Normal-to-Normal; SDNN = standard deviation of the NN interval; rMSSD = Root Mean Square of the Successive Differences; pNN50 = proportion of adjacent NN intervals which differ by more than 50 ms; VLF Power = Very Low Frequency *Power*; LF Power = Low Frequency Power; HF Power = High Frequency Power; LF/HF = Low Frequency Power/ High Frequency Power. Data presented as mean (SD), median (interquartile range)

^a^ = variables with non-normal distribution;

^b^ = Day recordings was considered when the Holter monitor was placed between 8:00 a.m. and 6:00 p.m;

**Fig 2:**
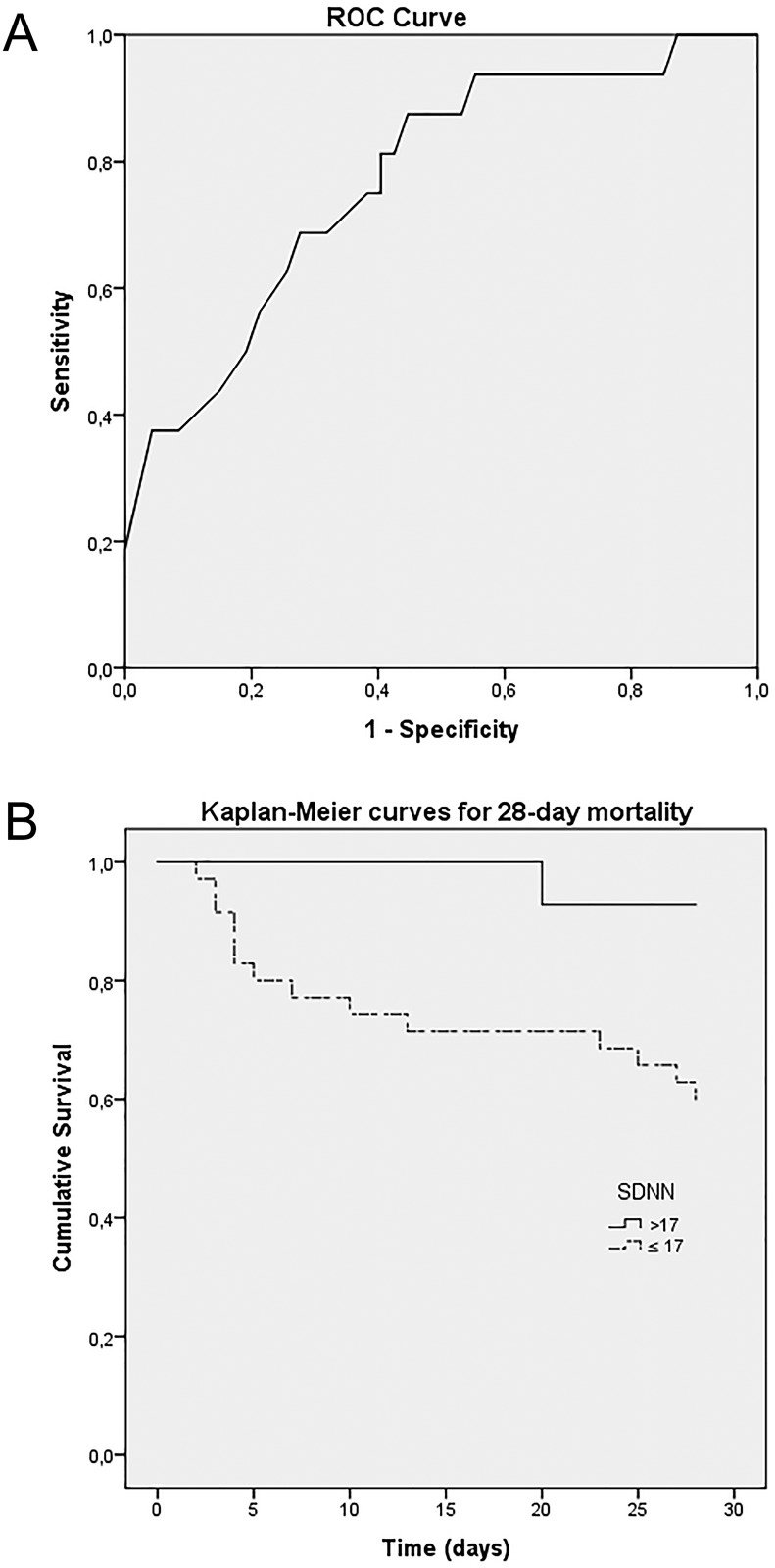
ROC Curve of SDNN and Kaplan-Meier curves for 28-day mortality. A: The ROC Curve of SDNN in 20-minute Holter in predicting 28-day mortality in patients with sepsis. The area under the curve was 0.772 (0.638–0.906). The value of 17ms was chosen as the cutoff point for SDNN (sensibility of 87.5%, specificity of 55.3%, positive likelihood ratio of 1.96 and negative likelihood ratio of 0.28). B: Kaplan-Meier curve showing 28-day mortality in septic patients with SDNN≤17ms (mean survival time of 21.3 days; 17.8–24.8) and SDNN>17ms (mean survival time of 27.4 days; 26.6–28.2). The survival curves were compared using log-rank test, p = 0.003, showing higher mortality in the patient group with SDNN≤17ms.

An unadjusted Cox regression for HRV parameters that were different between the two groups was built. It can be seen in Table 3. Since SDNN reached the larger difference between survivors and nonsurvivors, ROC curve was built to evaluate the accuracy of this parameter to predict the 28-day all-cause-mortality; as depicted in Fig 2, an area under the curve of 0.772 (0.638–0.906) was found. Then, because it presents the best relationship between sensitivity and specificity, 17ms was chosen as the cutoff point for SDNN. In order to test the possible clinical application of this cut-off point as a predictor of mortality in sepsis, patients were divided into two groups (SDNN> 17ms and SDNN≤17ms). As can be seen in Table 4, there is no significant difference between the baseline features of these two groups. Kaplan-Meier curve of these two groups (Fig 2) found log rank p = 0.003, showing higher mortality of the patient group with SDNN≤17ms. For the analysis of 28 days mortality, Cox regression for this dichotomous variable was made adjusted by the SOFA showing HR 6.3 (1.4–28.0; p = 0.015) for SDNN≤17ms and HR 1.3 (1.1–1.4; p = 0.001) for SOFA. Following a similar trend, Cox regression for dichotomous SDNN adjusted by the APACHE II showed HR 5.5 (1.2–24.8; p = 0.027) for SDNN≤17ms and HR 1.1 (1.02–1.12; p = 0.004) for APACHE II.

**Table 3:** Cox regression for heart rate variability parameters in 20-minute Holter.

Parameter	HR	95% CI	p-Value
**SDNN (ms)**	0.937	0.883–0.995	0.033
**Total Power (ms^2^)**	0.999	0.997–1.001	0.273
**VLF Power (ms^2^)**	0.998	0.996–1.001	0.269
**LF Power (ms^2^)**	0.998	0.993–1.003	0.352
**LF/HF**	0.619	0.380–1.009	0.054

HR = Hazard ratio; SDNN = standard deviation of the NN interval; VLF Power = Very Low Frequency *Power*; LF Power = Low Frequency Power; LF/HF = Low Frequency Power/ High Frequency Power

**Table 4:** Baseline characteristics of groups of SDNN≤17ms and SDNN>17ms.

Attribute	SDNN≤17 (n = 35)	SDNN>17 (n = 28)	p-Value
Age (y), SD	55 (20)	49 (17)	0.288
Male gender, %	22 (62.9)	16 (57.1)	0.796
APACHE II, SD	17.4 (8.16)	14.54 (6.16)	0.129
SOFA, SD	8.31 (3.68)	7.25 (3.43)	0.244
Mechanical Ventilation, %	22 (62.9)	14 (50.0)	0.306
**Underlying disease, n (%)**			
Cirrhosis	0 (0.0)	3 (10.7)	0.183
Dialytic patients	4 (11.4)	1 (3.6)	0.371
Hypertension	16 (45.7)	11 (39.3)	0.337
Diabetes	10 (28.6)	5 (17.9)	0.380
Stroke	4 (11.4)	5 (17.9)	0.192
Peripheral arterial disease	1 (2.86)	0 (0.0)	1.00
Heart Failure^b^	4 (11.4)	3 (10.7)	0.660
Coronary artery disease	6 (17.1)	1 (3.6)	0.234
Neoplasia	2 (5.7)	4 (14.3)	0.350
Chronic Obstructive Pulmonary Disease	2 (5.7)	1 (3.6)	0.899
Smoking	6 (17.1)	9 (32.1)	0.388
**Laboratory data**			
HB (g/dL)	10.3 (2.6)	9.9 (1.6)	0.528
White blood cells (per mm3)	14882 (7918)	18787 (11314)	0.113
Platelet × 103	205 (112)	233 (116)	0.365
Lactate^a^ (mmol/L)	1.70 (1.2–2.8)	2.0 (1.15–2.83)	0.787
C-reactive protein (mg/L)	276 (100)	204 (124)	0.013
Urea (mg/dL)	73.1 (40.7)	63.0 (48.1)	0.382
Creatinine^a^ (mg/dL)	1.27 (0.67–2.55)	0.71 (0.49–1.68)	0.223
Creatinine clearance^a^ (mL/min)	77 (28–125)	106 (51–154)	0.307
Glucose (mg/dL)	158 (78)	141 (57)	0.322
International normalized ratio^a^	1.24 (1.10–1.43)	1.29 (1.13–1.59)	0.302

Data presented as mean (SD), median (interquartile range) or absolute number (percentage).

^a^ = variables with non-normal distribution;

^b^ = Heart Failure was defined as previous echocardiogram with ejection fraction ≤ 50%.

Considering the small sample, we used modeling strategies to avoid the risk of overfitting and the excessive “optimism”. For the model with SOFA and dichotomous SDNN, optimism was calculated at 0.1075 (and the shrinkage factor was 0.8925), calculated HR 5.2 (1.2–23.0) for SDNN ≤17, with p = 0.03. For the model with APACHEII and dichotomous SDNN, optimism was calculated at 0.1834 (and the shrinkage factor was 0.8166), calculated HR 4.0 (0.9–18.1) for SDNN ≤17, with p = 0.07.

Finally, the calibration of the models with only SOFA and with dichotomized SDNN + SOFA was assessed with the the GF Test, showing, for the model with SOFA, p = 0.550, and, for the model with SOFA + SDNN, p = 0.600, indicating that there are no calibration problems. The GF test was valid under the usual assumption of proportional hazards of the Cox model. This assumption was not violated in the models considered, since the global risk proportionality test found p = 0.463 for the SOFA model only and p = 0.633 for the model With SOFA + SDNN. The addition of the dichotomized SDNN to the SOFA model increased the concordance statistic from 0.725 to 0.805 and the R^2^ of the model changed from 0.167 to 0.277. Furthermore, the AIC for the first model [SOFA] was 119.07 versus 112.17 for the second [SDNN + SOFA]. Greater values for concordance and R^2^ indicate a better model while smaller values for AIC indicate a better model. In order to evaluate the reclassification of the SOFA + SDNN model compared to the SOFA model only, we set the time of 28 days for the predictions of the risks, and calculated IDI (0.122; CI 0.043–0.235, p = 0.00) and NRI (0.408; CI 0.168–0.643, p = 0.01). These results suggest significant gains in the reclassification with the inclusion of SDNN in the model. All statistical analysis with the dichotomous SDNN can be seen in the Table 5.

**Table 5:** statistical analysis with the dichotomous SDNN.

**COX regression adjusted by SOFA**				
**Variable**		**HR**	**CI**	**p-Value**
SDNN≤17ms		6.3	1.4–28.0	0.015
SOFA		1.3	1.1–1.4	0.001
**COX regression adjusted by APACHE II**				
**Variable**		**HR**	**CI**	**p-Value**
SDNN≤17ms		5.5	1.2–24.8	0.027
APACHE II		1.1	1.02–1.12	0.004
**Model with dichotomous SDNN and SOFA optimism adjusted**				
**Variable**		**HR**	**CI**	**p-Value**
SDNN≤17ms		5.2	1.2–23.0	0.03
**Model with dichotomous SDNN and APACHE II optimism adjusted**				
**Variable**		**HR**	**CI**	**p-Value**
SDNN≤17ms		4.0	0.9–18.1	0.07
**Models of mortality prediction**				
**Model**	**GF Test**	**Concordance**	**R**^**2**^	**AIC**
SOFA	p = 0.550	0.725	0.167	119.07
SOFA + SDNN≤17ms	p = 0.600	0.805	0.277	112.17

HR = Hazard Ratio; CI = Confidence Interval; R^2^ = Explained variation; AIC = Akaike Information Criterion. For the analysis of 28 days mortality, Cox regression for dichotomous SDNN was made adjusted by the SOFA and adjusted by APACHE II. For the model with SOFA and dichotomous SDNN, optimism was calculated at 0.1075 (and the shrinkage factor was 0.8925). For the model with APACHEII and dichotomous SDNN, optimism was calculated at 0.1834 (and the shrinkage factor was 0.8166). The calibration of the models with only SOFA and with dichotomized SDNN + SOFA was assessed with the the GF Test, indicating that there are no calibration problems. The performance of those prediction models was assessed. Greater values for concordance and R^2^ indicate a better model while smaller values for AIC indicate a better model. In order to evaluate the reclassification of the SOFA + SDNN model compared to the SOFA model only, we calculated IDI (0.122; CI 0.043–0.235, p<0.01) and NRI (0.408; CI 0.168–0.643, p = 0.01). These results suggest significant gains in the reclassification with the inclusion of SDNN in the model.

In the survivor group, seven patients had undetermined infection source, while zero patients had undetermined infection source among the non-survivors. The results regarding the association of SDNN values and the outcome remained unchanged in the analysis excluding these seven patients. Thus, SDNN value was significantly higher among survivors as compared to non-survivors, when evaluated in the 20-minute Holter: 18.50 (10.00–34.50) and 8.50 (5.00–14.50), respectively, with p = 0.003. Cox regression for dichotomous SDNN adjusted by the SOFA or APACHE II revealed similar results (HR 7.1 [1.6–32.8]; p = 0.012, for SDNN≤17ms and HR 1.3 [1.1–1.5]; p < 0.001, for SOFA. Following a similar trend, Cox regression for dichotomous SDNN, adjusted by the APACHE II, showed HR 5.1 (1.1–22.9; p = 0.033) for SDNN≤17ms, and HR 1.1 (1.03–1.12; p = 0.001) for APACHE II.

## Discussion

In this prospective study with septic patients, we found that several HRV parameters obtained in the 20-minute Holter were correlated to 28-day all-cause mortality. In particular, SDNN ≤17 is associated with increased risk of death even after adjustment to SOFA or APACHE II. In contrast, HRV parameters in 24-hour Holter were not correlated to 28-day all-cause mortality.

Normal immune and physiologic responses eradicate pathogens through complex process involving generation of proinflammatory and anti-inflammatory mediators. The pathophysiology of sepsis is due to the inappropriate regulation of these normal reactions that becomes generalized and deleterious[25]. The role of the autonomic nervous system has been increasingly studied in the context of sepsis. Animal model studies suggest that vagus nerve stimulation increases the secretion of corticotropin-releasing hormone (CRH), ACTH, and cortisol[26]. Likewise, vagotomy attenuated fever response and corticosterone response produced by cytokines[27]. Acetylcholine, the principle vagal neurotransmitter, has an anti-inflammatory effect, attenuating the release of cytokines TNF, IL-1beta, IL-6 and IL-18 and preventing the development of shock[28]. Treatment with nicotine, a selective cholinergic agonist, and with choline, a precursor in the biosynthesis of acetylcholine, improved survival in experimental models of sepsis[29, 30]. This results supports that vagal afferent pathway are involved in peripheral cytokine-to-brain communication.

Several methods have already been developed to evaluate the autonomic function. Some of the tests would not be adequate for this study because they require active participation of patients. This is the case of the Valsalva’s manoeuvre, the deep breathing method, the isometric handgrip test, the mental arithmetic, and the active standing methods [31]. Other methods require infusion of drugs (e.g. baroreflex sensitivity testing with intravenous administration of phenylephrine), which could interfere in the treatment of patients with sepsis, making its use unfeasible and potentially harmful[31]. The serum catecholamines dosage can be used to evaluate the autonomic nervous system; however it has some limitations, providing information about the global autonomic function and not about organ-specific sympathetic function[32]. Additionally, the plasma concentration of norepinephrine, for example, depends not only on sympathetic activity, but also on norepinephrine reuptake and noradrenaline clearance from circulation [33]. Finally, patients with septic shock often receive external noradrenaline infusion as treatment. HRV is one of the most popular methods used to evaluate the autonomic function, presenting the advantages of being non-invasive and the fact that there are many commercial devices that provide the automated measurement of HRV[4].

The mechanism by which HRV is reduced in septic patients is not yet fully understood. In addition to the participation of the autonomic nervous system, recent studies in animals and cell cultures have shown that Lipopolysaccharides (amphiphilic components of the outer wall of Gram-negative bacteria) act in two ways on the hyperpolarization-activated cyclic nucleotide-gated channel 2 (HCN) of the atrial cells: directly inhibiting HCN-channels and indirectly sensitizing HCN-channels for sympathetic activation[34, 35].

Although there are no reference ranges of HRV parameters globally accepted, this study suggests that septic patients have reduced HRV compared to the general population. For example, in this study, the SDNN mean for surviving patients were 19.0ms and for nunsurviving patients were 8.5ms, while Kim et al found SDNN mean of 39.6ms for normal Korean Population[36].

The physiological meaning of each HRV parameter is very complex and not fully known. SDNN reflects all the cyclic components responsible for HRV (including sympathetic and parasympathetic activity) and is the most commonly used parameter[4]. HF Power reflects the vagal activity (parasympathetic) on the sinus node[37]. LF Power reflects the sympathetic and parasympathetic activity, with alleged predominance of the first [38]. The LF/HF ratio, in HRV, was classically described as an index of the sympathetic/parasympathetic balance[38]. However, several studies have shown that this interpretation is imprecise and simplistic and that the physiological meaning of this ratio remains controversial[39]. A reduced LF/HF ratio is associated with an increased risk of death in septic patients[40]. In this study, nonsurviving patients had lower Total Power, VLF Power, LF Power and LF / HF ratio than survivors. This finding is similar to that found in previous research[10, 12, 41].

Unlike the study by Duke et al.[13], in ours, HRV parameters were significantly different between survivors and nonsurvivors only in the 20-minute Holter. Holter with shorter periods of record is potentially more useful for be used in critical care patients, including those with sepsis, because these patients present immediate risk of death and therefore need a fast tool for definition of severity. Moreover, in such a dynamic condition as sepsis, a long time recording may suffer interference from therapeutic measures instituted, which can partially explain the negative results found with the 24-hour Holter in this study.

Global HRV parameters such as SDNN and TP were lower in nonsurviving patients of this study, which is consistent with the findings of previous studies[12, 13]. Chen et al[12] had demonstrated that SDNN would be a significant independent variable in the prediction of in-hospital mortality for emergency department patients with sepsis, although these authors did not present cut-off point for this HRV parameter. The cut-off of 17ms for SDNN obtained in a short time Holter record found in our study of might represent a useful tool due to identify patients with higher risk of death among septic patients in the daily practice. It worth mentioning that this result was maintained after adjustment for APACHE II or SOFA, indicating that this value could be an independent risk factor for mortality. Although the results found in the present study are statistically significant, the fact that the confidence intervals on the hazard ratio for SDNN are large reflect the small number of patients in our study, which indicates the need of confirming these results in larger series of septic patients.

Furthermore, concordance measure, R^2^, AIC, IDI and NRIindicate that predictive power of the SDNN + SOFA model is better than predictive power of SOFA only, which reinforces the possible clinical utility of this measure.

### Study limitations

The small number of patients is the main limitation of this study. In order to minimize this problem, it was used advanced modeling techniques to avoid the risk of overfitting and also to adjust the coefficients for optimism. This analysis kept SDNN ≤17 as a risk factor for death for the model with SOFA but not for the model with APACHE II. Another limitation of this study is the possible influence of other clinical conditions known to affect HRV as congestive heart failure, coronary artery disease, diabetes or mechanical ventilation use[4]. However, there was no difference between nonsurvivor and survivor groups about the frequency of these comorbidities (Table 1). Body temperature and medications (e.g., sedatives, beta-blockers, inotropic drugs) that can affect HRV were not evaluated in this study. Day-night variation in heart rate variability was not considered in the design of this study, although its existence has already been demonstrated in healthy volunteers with endotoxaemia[42]. The majority of 20-minute Holter measures were made during the day, and there were no significant differences between the percentage of day recordings from surviving and non-surviving groups. Furthermore, we do not know whether this day-night difference occurs in ICU patients. Finally, all included patients were enrolled before the publication of the Sepsis 3 Consensus[18], reason for which we were not able to use the new definitions of sepsis in the present study. Despite this, all patients included in this study had a SOFA score ≥ 2 points and met criteria for Sepsis based on this new consensus in a post hoc analysis.

Considering the small number of patients in this single-center study, we believe that the results found here are preliminary, hinting at the potential predictive capability of a dichotomized SDNN, what should be confirmed in future studies through external validation of the results in a separate population.

## Conclusions

Several HRV parameters are reduced in nonsurviving septic patients. Although further studies are necessary to confirm this finding, SDNN ≤17 is suggested as an independent risk factor for death in septic patients.

## References

[1] Walkey AJ, Wiener RS, Lindenauer PK. Utilization patterns and outcomes associated with central venous catheter in septic shock: a population-based study. Crit Care Med. 2013;41(6):1450–7. Epub 2013/03/20. . 23507718 10.1097/CCM.0b013e31827caa89 PMC3780984

[2] Fleischmann C, Scherag A, Adhikari NK, Hartog CS, Tsaganos T, Schlattmann P, et al Assessment of Global Incidence and Mortality of Hospital-treated Sepsis. Current Estimates and Limitations. Am J Respir Crit Care Med. 2016;193(3):259–72. Epub 2015/09/29. doi: 10.1164/rccm.201504-0781OC . 26414292 10.1164/rccm.201504-0781OC

[3] Pavon A, Binquet C, Kara F, Martinet O, Ganster F, Navellou JC, et al Profile of the risk of death after septic shock in the present era: an epidemiologic study. Crit Care Med. 2013;41(11):2600–9. Epub 2013/08/22. . 23963127 10.1097/CCM.0b013e31829a6e89

[4] Heart rate variability: standards of measurement, physiological interpretation and clinical use. Task Force of the European Society of Cardiology and the North American Society of Pacing and Electrophysiology. Circulation. 1996;93(5):1043–65. Epub 1996/03/01. . 8598068

[5] Sassi R, Cerutti S, Lombardi F, Malik M, Huikuri HV, Peng CK, et al Advances in heart rate variability signal analysis: joint position statement by the e-Cardiology ESC Working Group and the European Heart Rhythm Association co-endorsed by the Asia Pacific Heart Rhythm Society. Europace. 2015;17(9):1341–53. Epub 2015/07/17. doi: 10.1093/europace/euv015 . 26177817 10.1093/europace/euv015

[6] Vanderlei LC, Pastre CM, Hoshi RA, Carvalho TD, Godoy MF. Basic notions of heart rate variability and its clinical applicability. Rev Bras Cir Cardiovasc. 2009;24(2):205–17. Epub 2009/09/22. . 19768301 10.1590/s0102-76382009000200018

[7] Aboab J, Polito A, Orlikowski D, Sharshar T, Castel M, Annane D. Hydrocortisone effects on cardiovascular variability in septic shock: a spectral analysis approach. Crit Care Med. 2008;36(5):1481–6. Epub 2008/04/25. doi: 10.1097/CCM.0b013e31816f48f2 . 18434902 10.1097/CCM.0b013e31816f48f2

[8] Annane D, Trabold F, Sharshar T, Jarrin I, Blanc AS, Raphael JC, et al Inappropriate sympathetic activation at onset of septic shock: a spectral analysis approach. Am J Respir Crit Care Med. 1999;160(2):458–65. Epub 1999/08/03. doi: 10.1164/ajrccm.160.2.9810073 . 10430714 10.1164/ajrccm.160.2.9810073

[9] Tang CH, Chan GS, Middleton PM, Savkin AV, Lovell NH. Spectral analysis of heart period and pulse transit time derived from electrocardiogram and photoplethysmogram in sepsis patients. Conf Proc IEEE Eng Med Biol Soc. 2009;2009:1781–4. Epub 2009/12/08. doi: 10.1109/IEMBS.2009.5334005 . 19964557 10.1109/IEMBS.2009.5334005

[10] Barnaby D, Ferrick K, Kaplan DT, Shah S, Bijur P, Gallagher EJ. Heart rate variability in emergency department patients with sepsis. Acad Emerg Med. 2002;9(7):661–70. Epub 2002/07/03. . 12093705 10.1111/j.1553-2712.2002.tb02143.x

[11] Papaioannou VE, Dragoumanis C, Theodorou V, Gargaretas C, Pneumatikos I. Relation of heart rate variability to serum levels of C-reactive protein, interleukin 6, and 10 in patients with sepsis and septic shock. J Crit Care. 2009;24(4):625 e1–7. Epub 2009/03/31. doi: 10.1016/j.jcrc.2008.11.010 . 19327330 10.1016/j.jcrc.2008.11.010

[12] Chen WL, Chen JH, Huang CC, Kuo CD, Huang CI, Lee LS. Heart rate variability measures as predictors of in-hospital mortality in ED patients with sepsis. Am J Emerg Med. 2008;26(4):395–401. Epub 2008/04/16. doi: 10.1016/j.ajem.2007.06.016 . 18410805 10.1016/j.ajem.2007.06.016

[13] Gomez Duque M, Enciso Olivera C, Pena Torres E, Segura Duran OD, Nieto Estrada VH. [ECAIS study: inadvertent cardiovascular adverse events in sepsis]. Med Intensiva. 2012;36(5):343–50. Epub 2012/01/06. doi: 10.1016/j.medin.2011.11.008 . 22217461 10.1016/j.medin.2011.11.008

[14] von Elm E, Altman DG, Egger M, Pocock SJ, Gotzsche PC, Vandenbroucke JP. The Strengthening the Reporting of Observational Studies in Epidemiology (STROBE) statement: guidelines for reporting observational studies. J Clin Epidemiol. 2008;61(4):344–9. Epub 2008/03/04. doi: 10.1016/j.jclinepi.2007.11.008 . 18313558 10.1016/j.jclinepi.2007.11.008

[15] Levy MM, Fink MP, Marshall JC, Abraham E, Angus D, Cook D, et al 2001 SCCM/ESICM/ACCP/ATS/SIS International Sepsis Definitions Conference. Crit Care Med. 2003;31(4):1250–6. Epub 2003/04/12. doi: 10.1097/01.CCM.0000050454.01978.3B . 12682500 10.1097/01.CCM.0000050454.01978.3B

[16] Bone RC, Sprung CL, Sibbald WJ. Definitions for sepsis and organ failure. Crit Care Med. 1992;20(6):724–6. Epub 1992/06/01. . 1597021 10.1097/00003246-199206000-00002

[17] Dellinger RP, Levy MM, Rhodes A, Annane D, Gerlach H, Opal SM, et al Surviving sepsis campaign: international guidelines for management of severe sepsis and septic shock: 2012. Crit Care Med. 2013;41(2):580–637. Epub 2013/01/29. . 23353941 10.1097/CCM.0b013e31827e83af

[18] Singer M, Deutschman CS, Seymour CW, Shankar-Hari M, Annane D, Bauer M, et al The Third International Consensus Definitions for Sepsis and Septic Shock (Sepsis-3). Jama. 2016;315(8):801–10. Epub 2016/02/24. doi: 10.1001/jama.2016.0287 . 26903338 10.1001/jama.2016.0287 PMC4968574

[19] Vincent JL, Moreno R, Takala J, Willatts S, De Mendonca A, Bruining H, et al The SOFA (Sepsis-related Organ Failure Assessment) score to describe organ dysfunction/failure. On behalf of the Working Group on Sepsis-Related Problems of the European Society of Intensive Care Medicine. Intensive Care Med. 1996;22(7):707–10. Epub 1996/07/01. . 8844239 10.1007/BF01709751

[20] Knaus WA, Draper EA, Wagner DP, Zimmerman JE. APACHE II: a severity of disease classification system. Crit Care Med. 1985;13(10):818–29. Epub 1985/10/01. . 3928249

[21] Zhao YD, Rahardja D, Qu Y. Sample size calculation for the Wilcoxon-Mann-Whitney test adjusting for ties. Stat Med. 2008;27(3):462–8. Epub 2007/05/10. doi: 10.1002/sim.2912 . 17487941 10.1002/sim.2912

[22] R Core Team. R: A language and environment for statistical computing. Vienna, Austria: R Foundation for Statistical Computing; 2016.

[23] Florkowski CM. Sensitivity, specificity, receiver-operating characteristic (ROC) curves and likelihood ratios: communicating the performance of diagnostic tests. Clin Biochem Rev. 2008;29 Suppl 1:S83–7. Epub 2008/10/15. . 18852864 PMC2556590

[24] Steyerberg EW. Clinical prediction models: a practical approach to development, validation, and updating. New York, NY: Springer; 2009 xxviii, 497 p. p.

[25] Stearns-Kurosawa DJ, Osuchowski MF, Valentine C, Kurosawa S, Remick DG. The pathogenesis of sepsis. Annu Rev Pathol. 2011;6:19–48. Epub 2010/10/05. doi: 10.1146/annurev-pathol-011110-130327 . 20887193 10.1146/annurev-pathol-011110-130327 PMC3684427

[26] Gaykema RP, Dijkstra I, Tilders FJ. Subdiaphragmatic vagotomy suppresses endotoxin-induced activation of hypothalamic corticotropin-releasing hormone neurons and ACTH secretion. Endocrinology. 1995;136(10):4717–20. Epub 1995/10/01. doi: 10.1210/endo.136.10.7664696 . 7664696 10.1210/endo.136.10.7664696

[27] Fleshner M, Goehler LE, Schwartz BA, McGorry M, Martin D, Maier SF, et al Thermogenic and corticosterone responses to intravenous cytokines (IL-1beta and TNF-alpha) are attenuated by subdiaphragmatic vagotomy. J Neuroimmunol. 1998;86(2):134–41. Epub 1998/07/15. . 9663558 10.1016/s0165-5728(98)00026-5

[28] Borovikova LV, Ivanova S, Zhang M, Yang H, Botchkina GI, Watkins LR, et al Vagus nerve stimulation attenuates the systemic inflammatory response to endotoxin. Nature. 2000;405(6785):458–62. Epub 2000/06/06. doi: 10.1038/35013070 . 10839541 10.1038/35013070

[29] Wang H, Liao H, Ochani M, Justiniani M, Lin X, Yang L, et al Cholinergic agonists inhibit HMGB1 release and improve survival in experimental sepsis. Nat Med. 2004;10(11):1216–21. Epub 2004/10/27. doi: 10.1038/nm1124 . 15502843 10.1038/nm1124

[30] Parrish WR, Rosas-Ballina M, Gallowitsch-Puerta M, Ochani M, Ochani K, Yang LH, et al Modulation of TNF release by choline requires alpha7 subunit nicotinic acetylcholine receptor-mediated signaling. Mol Med. 2008;14(9–10):567–74. Epub 2008/06/28. doi: 10.2119/2008-00079.Parrish . 18584048 10.2119/2008-00079.Parrish PMC2435495

[31] Zygmunt A, Stanczyk J. Methods of evaluation of autonomic nervous system function. Arch Med Sci. 2010;6(1):11–8. Epub 2010/03/01. doi: 10.5114/aoms.2010.13500 . 22371714 10.5114/aoms.2010.13500 PMC3278937

[32] AKSMSBIM C.. Sistema Nervoso Autônomo e Doença Cardiovascular. Revista da Sociedade de Cardiologia do Rio Grande do Sul. 2004;03:1–7.

[33] Esler M, Jennings G, Lambert G, Meredith I, Horne M, Eisenhofer G. Overflow of catecholamine neurotransmitters to the circulation: source, fate, and functions. Physiol Rev. 1990;70(4):963–85. Epub 1990/10/01. . 1977182 10.1152/physrev.1990.70.4.963

[34] Ebelt H, Geissler I, Ruccius S, Otto V, Hoffmann S, Korth H, et al Direct inhibition, but indirect sensitization of pacemaker activity to sympathetic tone by the interaction of endotoxin with HCN-channels. Clin Exp Pharmacol Physiol. 2015;42(8):874–80. Epub 2015/05/02. doi: 10.1111/1440-1681.12415 . 25933122 10.1111/1440-1681.12415

[35] Klockner U, Rueckschloss U, Grossmann C, Ebelt H, Muller-Werdan U, Loppnow H, et al Differential reduction of HCN channel activity by various types of lipopolysaccharide. J Mol Cell Cardiol. 2011;51(2):226–35. Epub 2011/05/26. doi: 10.1016/j.yjmcc.2011.05.004 . 21609720 10.1016/j.yjmcc.2011.05.004

[36] Kim GM, Woo JM. Determinants for heart rate variability in a normal Korean population. J Korean Med Sci. 2011;26(10):1293–8. Epub 2011/10/25. doi: 10.3346/jkms.2011.26.10.1293 . 22022180 10.3346/jkms.2011.26.10.1293 PMC3192339

[37] Lahiri MK, Kannankeril PJ, Goldberger JJ. Assessment of autonomic function in cardiovascular disease: physiological basis and prognostic implications. J Am Coll Cardiol. 2008;51(18):1725–33. Epub 2008/05/03. . 18452777 10.1016/j.jacc.2008.01.038

[38] Pagani M, Lombardi F, Guzzetti S, Sandrone G, Rimoldi O, Malfatto G, et al Power spectral density of heart rate variability as an index of sympatho-vagal interaction in normal and hypertensive subjects. J Hypertens Suppl. 1984;2(3):S383–5. Epub 1984/12/01. . 6599685

[39] Billman GE. The LF/HF ratio does not accurately measure cardiac sympatho-vagal balance. Front Physiol. 2013;4:26 Epub 2013/02/23. doi: 10.3389/fphys.2013.00026 . 23431279 10.3389/fphys.2013.00026 PMC3576706

[40] Korach M, Sharshar T, Jarrin I, Fouillot JP, Raphael JC, Gajdos P, et al Cardiac variability in critically ill adults: influence of sepsis. Crit Care Med. 2001;29(7):1380–5. Epub 2001/07/11. . 11445691 10.1097/00003246-200107000-00013

[41] Tateishi Y, Oda S, Nakamura M, Watanabe K, Kuwaki T, Moriguchi T, et al Depressed heart rate variability is associated with high IL-6 blood level and decline in the blood pressure in septic patients. Shock. 2007;28(5):549–53. Epub 2007/12/14. doi: 10.1097/shk.0b013e3180638d1 . 18075483 10.1097/shk.0b013e3180638d1

[42] Alamili M, Rosenberg J, Gogenur I. Day-night variation in heart rate variability changes induced by endotoxaemia in healthy volunteers. Acta Anaesthesiol Scand. 2015;59(4):457–64. Epub 2015/03/20. doi: 10.1111/aas.12472 . 25790066 10.1111/aas.12472

